# Isolation and Identification of G8P[1] Bovine Rotavirus A Among Neonatal Diarrheic Calves in Yunnan, China

**DOI:** 10.3390/ani16081274

**Published:** 2026-04-21

**Authors:** Peiying Zhu, Yan Liu, Muhammad Khan, Hongmei Liu, Veerasak Punyapornwithaya, Chenxi Zhang, Xin Wu, Hongya Yan, Huafeng Gao, Wengui Li

**Affiliations:** 1 Joint International R&D Center of Veterinary Public Health, College of Veterinary Medicine, Yunnan Agricultural University, Kunming 650201, China; 2 Faculty of Veterinary Medicine, Khon Kaen University, Khon Kaen 40002, Thailand; 3 Yunnan Academy of Animal Husbandry and Veterinary Science, Kunming 650224, China; khanbwn011@gmail.com (M.K.);; 4 Faculty of Veterinary Medicine, Chiang Mai University, Chiang Mai 50100, Thailand

**Keywords:** calf, rotavirus A, G8P[1] strain, whole-genome analysis, reassortment

## Abstract

Bovine rotavirus (BRV) poses a major threat to the global cattle industry, driving significant morbidity and mortality in young calves. In Yunnan Province, China, BRV is the primary cause of neonatal calf diarrhea (NCD), yet the molecular epidemiology of circulating strains remains poorly understood. This study aimed to investigate the molecular characteristics of bovine rotavirus strains associated with a severe outbreak of the NCD on a local farm. Fecal samples were collected from 396 calves and screened for BRV by RT-PCR targeting the VP6 gene. Positive samples were subjected to virus isolation in MA104 cells, followed by whole-genome sequencing, phylogenetic analysis, and pathogenicity assessment in suckling mice. Of 396 samples, 85 tested positive for BRV, corresponding to an animal-level prevalence of 21.5% (95% CI: 17.5–25.8%), with four fatalities recorded. A strain designated as BRV-YN1-2021 was successfully isolated, exhibiting characteristic cytopathic effects, specific immunofluorescence, and typical rotavirus morphology by electron microscopy. Genomic analysis revealed the constellation G8-P[1]-I2-R2-C2-M2-A3-N2-T6-E2-H3, identified as genotype G8P[1]. BLAST analysis showed that four genomic segments shared the highest identity with deer rotavirus strains, five with human rotavirus strains, and two with bovine rotavirus strains. Phylogenetic analysis demonstrated close relationships with US deer strains, Japanese bovine strains, and human strains circulating in China. Experimental infection in suckling mice induced diarrhea and significant intestinal histopathology, degeneration of villous epithelial cells, goblet cell hyperplasia, and inflammatory infiltration. This study reports the first isolation of a G8P[1] bovine rotavirus from a diarrhea outbreak in Chinese cattle. The multi-host genetic composition provides evidence of interspecies reassortment events, highlighting the zoonotic potential of BRV and emphasizing the need for continuous molecular surveillance to inform effective control strategies.

## Simple Summary

Viral diarrhea, particularly that associated with rotavirus type A, is one of the leading causes of early calf mortality and thus poses a challenge to the sustainability of the cattle industry worldwide. This study investigated bovine rotavirus in a total of 396 calves during a severe diarrhea outbreak on a dairy farm in Yunnan, China. A novel rotavirus strain was detected and isolated, and genetic analysis confirmed it as G8P[1] with a unique genomic composition that differed from previously reported bovine rotavirus strains in China, suggesting possible genetic assortment or the circulation of unrecognized strains in cattle. Overall, this study provides basic understanding of bovine rotavirus diversity in Yunnan Province, China, and highlights the need for continued surveillance to support effective disease control in cattle herds.

## 1. Introduction

Rotavirus (RV) belongs to the family Reoviridae, subfamily Sedoreovirinae, and genus rotavirus. It is a non-enveloped virus measuring approximately 60–80 nm in diameter, with a genome of about 18.5 kb [1]. Rotaviruses infect a broad range of mammalian and avian hosts, and infections are generally host-restricted [2]. Based on the classification of the International Committee on Taxonomy of Viruses, rotaviruses are currently divided into nine species (Rotavirus A–D and F–J). Among these, species A, B, and C are known to infect both humans and animals, with species A being the most prevalent. Rotavirus A (RVA) strains are typically designated according to their host origin, such as bovine (BRV), porcine (PRV), and rabbit (RRV) rotaviruses [3,4].

BRV is prevalent in global cattle-raising regions, causing substantial economic losses to the cattle industry [5,6]. It can cause depression, acidosis, anorexia, and watery diarrhea in newborn calves. This virus has a brief incubation period, especially in high-risk areas, elucidating the ecological requirements of the vectors, and supporting preparedness efforts impacting animals aged 1 to 7 days, and is characterized by severe diarrhea and dehydration [7]. The incidence rate for this age group is between 60% and 80%, with mortality rates potentially as high as 30% [8]. When calves suffer secondary or co-infections from pathogens such as bovine coronavirus or Escherichia coli, their clinical signs will become more severe, potentially leading to death in extreme cases [9]. BRV group A is recognized as the leading cause of severe gastroenteritis in cattle, causing high morbidity and mortality rates in newborn calves, and consequently leading to significant economic losses [5,6].

The RVA genome is composed of 11 double-stranded RNA (dsRNA) segments, which encode six structural proteins (VP1–VP4, VP6, and VP7) and six nonstructural proteins (NSP1–NSP6). Each segment contains both 5′- and 3′-untranslated regions (UTRs) [10]. The VPs constitute the infectious triple-layered particles enveloping the genomic dsRNA. NSPs are chiefly responsible for dsRNA replication and transcription, cellular pathogenesis, and viral particle maturation [11]. Research has shown that during rotavirus invasion of host cells, the VP4 protein is hydrolyzed by trypsin into two peptides, VP5 and VP8 [12]. VP5 (60 kDa) remains non-covalently associated with the infectious particle, initiating the entry process. The VP8 (28 kDa) subunit exhibits hemagglutinin activity, engages in host sialic acid binding, and is crucial for virus tropism. VP4 harbors the main antigenic sites, which are vital for vaccine development. The enzymatic activation of VP4 by pancreatic trypsin significantly enhances BRV pathogenicity [13]. The VP6 protein is commonly used to differentiate rotavirus species based on their antigenic and genetic characteristics [14].

The classification of RVA mainly focuses on two viral surface proteins: VP7, which determines the G (glycoprotein) genotype, and VP4, which defines the P (protease-sensitive protein) genotype [15,16]. To date, at least 12 distinct G genotypes (G1–G3, G5, G6, G8, G10, G11, G15, G17, G21, and G24) and 11 different P genotypes (P[1], P[3], P[5]–P[7], P[11], P[14], P[17], P[21], P[29], and P[38]) have been identified in cattle. Among these, G6, G8, G10, P[1], P[5], and P[11] are the most prevalent genotypes globally, as shown in previous research [3,17]. In China, several G genotypes (G6, G8, and G10) and P genotypes (P[1], P[5], P[7], and P[11]) have been detected in cattle. Notably, a previous study in multiple provinces of China (Shandong, Liaoning, Henan, Xinjiang, Shanxi, and Jilin) found G6 and P[5] to be the predominant G and P genotypes [18], respectively, with the G6P[5] combination being the most common. Additionally, G10 has been frequently identified in some areas. The association of G and P genotypes with their host species is a common phenomenon due to host-specific barriers and limitations on rotavirus infection. However, atypical G and P genotypes of human rotaviruses have emerged from interspecies transmission and natural genetic reassortment between human and animal viruses, particularly those from cattle and pigs [19].

BRV is recognized as one of the primary viral agents causing diarrhea in calves and has substantial economic impacts on the cattle industry in China [6]. Epidemiological studies and meta-analyses indicate that BRV infection is common among cattle populations, with pooled prevalence estimates ranging from approximately 35% to 46% across various regions and age groups in mainland China, underscoring its widespread circulation among bovine herds [20]. In addition to BRV mono-infections, co-infections with other enteric pathogens such as bovine coronavirus (BCoV), bovine viral diarrhea virus (BVDV), noroviruses, and neboviruses have been frequently reported in diarrheic calves, highlighting the complexity of viral interactions and challenges in disease diagnosis and control [21,22,23]. These mixed infections can complicate clinical outcomes and may contribute to the increased severity and persistence of diarrhea in affected herds. Nevertheless, epidemiological and molecular data on BRV in the southwestern region of China remain limited. Therefore, this study aimed to determine the molecular epidemiology of the BRV in the outbreak farm using RT-PCR and viral isolation techniques. This effort is critical for gaining insights into the prevalence, genetic diversity, and the emergence of new pathogenic strains of this virus, which are crucial for developing effective prevention and control strategies for curbing the spread of BRV among cattle populations.

## 2. Materials and Methods

### 2.1. Ethical Approval and Sample Collection

All procedures and protocols of this study were in accordance with and approved by the Animal Ethics Committee of Yunnan Agricultural University (APYNAU202503090). Twenty 3-week-old SPF Kunming mice from the Experimental Animal Center of Kunming Medical University were acclimatized under standard laboratory conditions for two weeks before use. This study was executed between 2020 and 2021, when a severe outbreak of illness was observed in neonatal calves (characterized by high fever of 39.2–41.1 °C with diarrhea) among three neighboring cattle farms in southwestern Yunnan Province. Total breeding scales of the three cattle farms were 1150, 685 and 489 heads, respectively, accounting for a combined total of 2324 cattle across all affected farms. A total of 396 calves were aged ≤6 months, making up 17% of the entire herd. Calves aged between a half month to three months were reported to have died due to diarrhea at three adjacent farms; calves of other ages primarily showed clinical signs of diarrhea. Fecal samples of calves were collected from the farms at the peak of the outbreak and the positive samples were detected by RT-PCR and verified as group A rotavirus infection by sequencing the amplified fragments.

In this survey, three farms operated through self-sustained breeding cycles, with only bulls introduced from a local breeding stock farm. Stamping-out strategies and vaccination against foot and mouth disease (FMD), as well as strict biosafety measures, have been employed. In addition, immunization against bovine viral diarrhea virus (BVDV) has also been completed. However, no licensed commercial vaccines against bovine rotavirus are available.

After vital procedures to ensure a suspected rotavirus disease outbreak, fecal samples were collected from 396 calves in 10 mL centrifuge tubes and immediately transported to the university lab by ensuring cold chain supply, and then stored at −80 °C until subsequent analysis. Additionally, fecal samples were collected from 10 calves with severe diarrhea for virus isolation.

The prevalence of BRV at the farm was calculated by using the following equation.

Prevalence = (number of calves positive to BRV/total calves in the farm) × 100%

Serum samples were also collected from BRV-positive calves for antibody preparation, which were subsequently used in immunofluorescence assays.

### 2.2. RT-PCR Analysis for Clinical Samples

Viral nucleic acids were extracted from the 396 fecal samples using the TaKaRaMiniBEST Viral RNA/DNA Extraction Kit (TaKaRa, Dalian, China) according to the manufacturer’s instructions. Extracted nucleic acids were stored at −80 °C until further analysis. Primers targeting the BRV VP6 gene were designed using Primer 5.0 software. The primer design was based on VP6 gene sequences obtained from a previously identified BRV-positive calf diarrhea sample in Yunnan, in addition to reference BRV sequences available in GenBank. The primer sequences were as follows: forward primer (F), 5′-TTCAGGTCGCTGGATTC-3′; and reverse primer (R), 5′-TGGAAATAATGCCGCTAC-3′. The expected amplicon size was 380 bp. Reverse transcription polymerase chain reaction (RT-PCR) was performed using the PrimeScript™ One-Step RT-PCR Kit (TaKaRa, Dalian, China). The thermal cycling conditions consisted of reverse transcription at 50 °C for 30 min and initial denaturation at 95 °C for 2 min, followed by 35 cycles of denaturation at 94 °C for 40 s, annealing at 55 °C for 30 s, and extension at 72 °C for 40 s. A final extension step was carried out at 72 °C for 7 min. Amplified products were analyzed by agarose gel electrophoresis, and the results were visualized using a UV gel imaging system.

### 2.3. Virus Isolation and Immunofluorescence Assay

Ten BRV-positive fecal samples from diarrheic calves were selected for virus isolation. The samples (10 mL) were diluted in 20 mL phosphate-buffered saline (PBS) and clarified by centrifugation at 3000 rpm for 30 min. The supernatants were subsequently harvested and filtered through a 0.45 μm membrane (Millipore, Billerica, MA, USA). An aliquot (10 mL) of the filtrate was incubated with 15 μg/mL TPCK-treated trypsin (Sigma-Aldrich, St. Louis, MO, USA) at 37 °C for 40 min [24]. Prior to inoculation, confluent MA104 cell monolayers were prepared by removing the culture medium and washing the cells with calcium- and magnesium-free PBS (Sigma-Aldrich) [25]. The treated supernatants were subsequently added to the cell monolayers and incubated at 37 °C in a humidified environment with 5% CO_2_. The cultures were observed daily for cytopathic effects (CPEs). To propagate the virus, infected cultures were subjected to freeze–thaw cycles, followed by re-treatment with trypsin, and used to infect fresh MA104 monolayers. Samples that failed to produce CPEs after six blind passages were regarded as negative. Successfully isolated virus was further amplified through six serial passages in MA104 cells, with continuous observation of CPE.

Virus titration was performed in 96-well plates using 10-fold serial dilutions. The presence of CPEs was evaluated under a microscope at 4 days post-inoculation, and viral titers were calculated using the Reed–Muench method, expressed as TCID_50_/0.1 mL.

To observe viral proliferation, MA104 cells were seeded in six-well plates and infected with the isolated strain, designated BRV-YN1-2021. Following infection, cells were fixed with cold acetone (−20 °C) for 20 min and washed three times with PBS (pH 7.2). The fixed cells were then incubated with BRV-positive bovine serum, which was obtained from recovered calves with blood collected 15 days after recovery, for 30 min at 37 °C, then stained with a 100-fold diluted fluorescein isothiocyanate (FITC)-conjugated rabbit anti-bovine antibody (BBI, Shanghai, China). After washing with PBS, the plates were air-dried and examined under a fluorescence microscope at 200× magnification.

### 2.4. Transmission Electron Microscopy

Following the six passages, BRV-infected cell cultures were prepared for transmission electron microscopy analysis. The culture medium was discarded, and the cell monolayers were washed once with pre-warmed culture medium of the same type. Cells were then scraped from the culture vessel, collected, and concentrated by low-speed centrifugation. The supernatant was discarded, and the cell pellet was resuspended at room temperature in electron microscopy fixative. The cell suspension was transferred to a 1.5 mL tube and centrifuged continuously at 10,000 rpm for 10 min. The supernatant was removed, and fresh electron microscopy fixative was gently added along the tube wall to avoid disturbing the pellet. Samples were stored at 4 °C until shipment to Sichuan Scientist Biotech Co., Ltd. (Chengdu, China). for processing and examination. Viral particles were visualized using a Hitachi 7100 transmission electron microscope (Hitachi, Tokyo, Japan).

### 2.5. Genomic Sequencing and Assembly

Viral nucleic acid was extracted from the isolated BRV strain, and the quality of the extracted genomic RNA was assessed. A complementary DNA (cDNA) library was prepared. For library construction, the viral RNA was fragmented to approximately 300–500 bp according to the manufacturer’s instructions. The fragmented RNA was then subjected to random reverse transcription, followed by synthesis of double-stranded cDNA. End-repair reactions were performed on both termini of the fragmented cDNA, and sequencing adapters were ligated. Sequencing was carried out using the Illumina NovaSeq 6000 PE150 platform (San Diego, CA, USA).

The obtained sequence reads were assembled into contigs using de novo assembly. The assembled contigs were used as query sequences in BLASTsearches using the online tool provided by the National Center for Biotechnology Information (NCBI) against the non-redundant nucleotide database to obtain the complete nucleotide sequences of each gene segment of the isolated BRV-YN1-2021 strain.

### 2.6. Genotyping and Phylogenetic Analyses

Genotyping of all 11 BRV genome segments was performed using the Rotavirus A genotyping tool available through the Bacterial and Viral Bioinformatics Resource Center (BV-BRC; https://www.bv-brc.org/ accessed on 10 January 2025). Putative open reading frames (ORFs) and their corresponding amino acid sequences were predicted using the ORF Finder tool (http://www.ncbi.nlm.nih.gov/gorf/gorf.html accessed on 21 March 2025). 

Phylogenetic analyses were conducted for the VP4, VP6, and VP7 genes, as well as for the complete genome of the isolated BRV strain. Phylogenetic trees were constructed using the maximum likelihood (ML) method with 1000 bootstrap replicates, implemented in MEGA version 6.06.

### 2.7. Animal Experimentation and Histopathological Analysis

As described by Rey et al. [26], specific pathogen-free Kunming mice (n = 18; age, 7 days) were randomly allocated into two groups (n = 9 per group) and housed in separate cages. Mice in the BRV infection group were orally gavaged with 0.1 mL of Dulbecco’s Modified Eagle Medium (DMEM) containing the BRV-YN1-2021 isolate at a titer of 10^5.17^ TCID_50_/mL. Mice in the control group received 0.1 mL of sterile DMEM by oral gavage. All animals were observed daily for clinical signs. In addition, fecal samples were collected from the mice and tested for the confirmation of BRV infection by RT-PCR.

Three mice from each group were humanely euthanized at 3, 6, and 9 days post-inoculation. Small intestine samples were collected from each animal and processed for histopathological analysis.

For histopathological analysis, tissue samples were fixed in 10% neutral buffered formalin for 48 h and then routinely processed through graded ethanol dehydration, xylene clearing, and paraffin embedding. Sections approximately 4–5 μm thick were cut using a microtome, mounted on glass slides, and stained with hematoxylin and eosin (H&E) using routine histological procedures. The stained sections were examined under a light microscope for characteristic pathological changes, including villous atrophy, crypt hyperplasia, epithelial cell desquamation, and inflammatory cell infiltration in the lamina propria.

### 2.8. Statistical Analysis

The 95% confidence interval for the prevalence estimate was calculated using R version 4.4.2 with the “prevalence” package (https://www.r-project.org/ accessed on 10 April 2025).

## 3. Results

### 3.1. Epidemiology Survey

A total of 396 fecal samples were collected from the affected farm and analyzed using a reverse transcription polymerase chain reaction (RT-PCR). Eighty-five samples tested positive for bovine rotavirus (BRV), corresponding to an animal-level prevalence of 21.5% (85/396; 95% confidence interval: 17.5–25.8%) on the outbreak farm. Additionally, among the BRV-positive calves, four died due to diarrhea-related complications.

### 3.2. Virus Isolation, Indirect Immunofluorescence Assay, and Transmission Electron Microscopy

**Figure 1:**
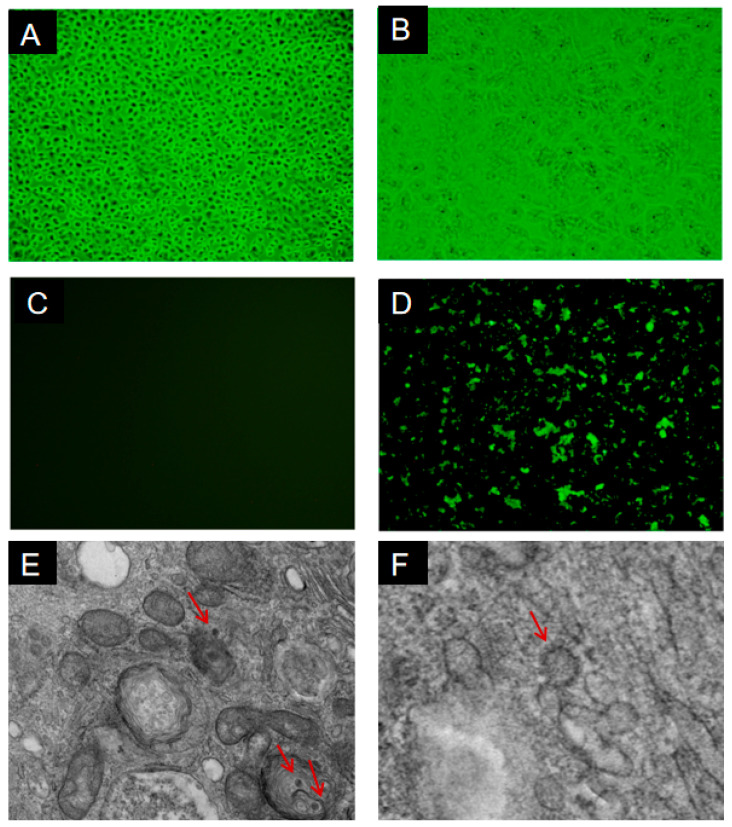
Isolation and characterization of BRV-YN1-2021 in MA104 cells. (**A**) Mock-infected control MA104 cells showing normal morphology. (**B**) Cytopathic effect (CPE) in MA104 cells at 4 days post-inoculation with BRV-YN1-2021, characterized by cell rounding, cytoplasmic granulation, and detachment (10 × 20). (**C**) Immunofluorescence assay of mock-infected control MA104 cells showing no specific fluorescence. (**D**) Immunofluorescence assay of MA104 cells infected with BRV-YN1-2021 at 4 days post-inoculation, showing specific fluorescence in the cytoplasm. (**E**,**F**) Negative staining transmission electron microscopy (TEM) images of BRV-YN1-2021 viral particles, approximately 80 nm in diameter, displaying characteristic wheel-like rotavirus morphology. Magnification: 150,000×. (Red arrow: rotavirus virions).

Fecal samples from calves that tested positive for BRV by RT-PCR were selected for virus isolation in cell culture. Following inoculation of MA104 cells, the appearance of a cytopathic effect (CPE) was evident through a series of characteristic cellular alterations. Initially, a notable increase in cytoplasmic granularity was observed, accompanied by the obscuration of cell boundaries. Subsequently, cells exhibited rounding, which progressed to degeneration and eventual detachment by the third passage. Stable and reproducible CPEs were consistently observed from passage six onward. These changes were characterized by clusters of enlarged, rounded cells with a densely granular appearance (Figure 1B), indicating successful viral replication and virus-induced disruption of normal cellular morphology and function. No CPE was observed in mock-infected control MA104 cells (Figure 1A).

To confirm efficient viral propagation, the BRV isolate was serially passaged in MA104 cells. At four days post-inoculation, cells infected with the isolate were fixed with cold acetone and stained using BRV-positive cattle serum, followed by FITC-conjugated secondary antibody. Specific fluorescence was detected in the cytoplasm of cells infected with BRV-YN1-2021 (Figure 1D), while no fluorescence was observed in mock-infected control cells (Figure 1C). The viral titer of the isolated strain was calculated as 10^5·17^ TCID_50_/mL. These results confirm the successful isolation of BRV strain BRV-YN1-2021 from diarrheic fecal samples collected in Yunnan Province during 2020–2021. Transmission electron microscopy (TEM) was employed to examine viral particles in infected cells following inoculation. The isolated strain exhibited characteristic rotavirus morphology, with viral particles measuring approximately 80 nm in diameter and displaying a distinct wheel-like surface structure, consistent with the typical morphology of bovine rotavirus (Figure 1E,F).

### 3.3. Genotype Constellation Analysis of BRV

We successfully isolated two strains of BRV from the positive samples, but sequencing analysis showed that the genetic sequences of the two strains were identical and both came from the same cattle farm. Therefore, we selected one of the strains as a representative virus and named it BRV-YN1-2021. The whole genome of this strain has been sequenced and submitted to the GenBank database with accession numbers PX551869–PX551879. Genotyping using the Rotavirus A genotyping tool revealed that the strain possesses the genotype constellation G8-P[1]-I2-R2-C2-M2-A3-N2-T6-E2-H3. Following the standardized rotavirus nomenclature guidelines, the strain was designated RVA/bovine/CHN/BRV-YN1-2021/2021/G8P[1].

BLAST analysis was performed to determine the nucleotide identities of all 11 genome segments of BRV-YN1-2021 against reference strains. The analysis revealed that four segments exhibited the highest similarity to deer rotavirus strains, five segments to human rotavirus strains, and two segments to bovine rotavirus strains. The lengths of the 11 gene segments, putative open reading frames (ORFs), and associated promoter and terminator signals were analyzed using the NCBI ORF Finder tool (Table 1).

Among the four segments most closely related to deer strains, the VP4, VP2, NSP1, and NSP3 gene fragments were 2331 bp, 2643 bp, 1476 bp, and 901 bp in length, respectively. Their nucleotide sequence identities ranged from 75.5 to 99.7%, 92.8–99.6%, 91.1–99.2%, and 93.2–99.5%, with corresponding amino acid identities of 57.0–99.7%, 43.8–100.0%, 50.7–99.8%, and 46.5–98.5%. The five segments highly similar to human strains corresponded to VP6, VP1, VP3, NSP2, and NSP4, with lengths of 1194 bp, 3267 bp, 2507 bp, 953 bp, and 527 bp, respectively. Nucleotide identities ranged from 95.1 to 99.3%, 93.8–97.2%, 92.0–96.7%, 93.1–98.7%, and 91.5–97.5%, while amino acid identities were 52.1–100.0%, 81.9–98.6%, 41.8–96.8%, 33.2–99.8%, and 49.7–97.9%. The VP7 segment (981 bp) was most closely related to a bovine rotavirus strain, with nucleotide and amino acid identities ranging from 96.3 to 97.7% and 71.2–99.1%, respectively. The NSP5 segment (557 bp) was phylogenetically closest to a bovine rotavirus strain, exhibiting a nucleotide identity of 95.5–99.8% and amino acid identity of 29.8–100% (Table 1). This genomic constellation suggests that BRV-YN1-2021 is a typical G8P[1] rotavirus assembled on a genetic backbone comprising Wa-like, AU-like, and 14-02218-2 genotypes. The observed segment-specific similarities to strains from different host species indicate that this strain likely emerged through multiple interspecies reassortment events (Table 2).

### 3.4. Genome Alignment of VP4 and VP7

The nucleotide sequences of the VP4 and VP7 gene segments of the BRV isolate BRV-YN1-2021 were determined and analyzed. The sequenced VP4 gene segment contained an open reading frame (ORF) of 2331 nucleotides (nt) encoding 777 amino acid residues, with no insertions or deletions detected. Comparative analysis of the nucleotide and deduced amino acid sequences with Chinese isolates and other reference strains available in GenBank revealed identities ranging from 49.6% to 99.7% at the nucleotide level and 32.0% to 99.7% at the amino acid level (Table 3).

The BRV-YN1-2021 strain exhibited the highest nucleotide identity (99.7%) with the cervid strain C/Cervidae/United States/14-02218-2/2014 (KU962131), which belongs to genotype G8P[1]. When compared with other G8P[1] strains, the isolate shared 96.9% nucleotide identity and 99.1% amino acid identity with the bovine isolate RVA/Bovine-tc/JPN/AH1041/2022. The BRV-YN1-2021 strain also demonstrated high nucleotide identity with the rhesus-derived strain RVA/Rhesus-tc/USA/PTRV/1990. Additionally, the BRV-YN1-2021 strain shares 94.4% nucleotide identity with the equine genotype G6P[1] strain RVA/Horse-wt/IND/ERV2/2015.

Among Chinese strains, the highest nucleotide identity was observed with the bovine isolate RVA/Cow-tc/CHN/SDC1/2018 (genotype G8P[1]). The lowest nucleotide identity was with the Korean swine isolate RVC/Pig-wt/KOR/07-109-12/2007 (genotype G6P[X]), with which BRV-YN1-2021 shared only 49.6% nucleotide identity and 32.0% amino acid identity.

The VP7 gene segment of BRV-YN1-2021 contained a 981 nt ORF encoding 327 amino acid residues, with no insertions or deletions detected. The nucleotide and deduced amino acid sequences exhibited similarities ranging from 48.2% to 97.7% and 31.6% to 99.1%, respectively (Table 3).

The BRV-YN1-2021 strain shared the highest nucleotide identity (97.7%) with the bovine isolate RVA/Cow-tc/JPN/GB12-22/2007 (LC553600), which belongs to genotype G8P[11]. The isolate demonstrated 96.9% nucleotide identity and 99.1% amino acid identity with the bovine isolate RVA/Bovine-tc/JPN/AH1041/2022, and comparable identities (96.9% nucleotide and 99.1% amino acid) with the cervid strain C/Cervidae/USA/14-02218-2/2014 (genotype G8P[1]). Among human strains, the highest nucleotide identity was observed with RVA/Human-wt/US/2012841174/2012 (KJ411440), which belongs to genotype G8P[14]. The lowest identities were with the US swine isolate RVC/Pig-wt/USA/OK.5.68/2008 (MH282893, genotype GX), with which BRV-YN1-2021 shared only 48.2% nucleotide identity and 31.6% amino acid identity.

Analysis of the VP4 and VP7 ORFs revealed that the Yunnan isolate exhibited the highest similarity to deer-derived isolates from the United States, followed by bovine-derived isolates from Japan. The genetic sequence data also indicated significant homology to human-derived and rhesus-derived strains, suggesting the potential occurrence of interspecies recombination events. Additionally, the close relationship between BRV-YN1-2021 and the Chinese bovine-derived strain RVA/Cow-tc/CHN/SDC1/2018 (genotype G6P[1]) indicates the circulation of diverse rotavirus genotypes among bovine herds in China.

### 3.5. Phylogenetic Analysis of Genes Encoding Structural Proteins VP4, VP6, and VP7

**Figure 2:**
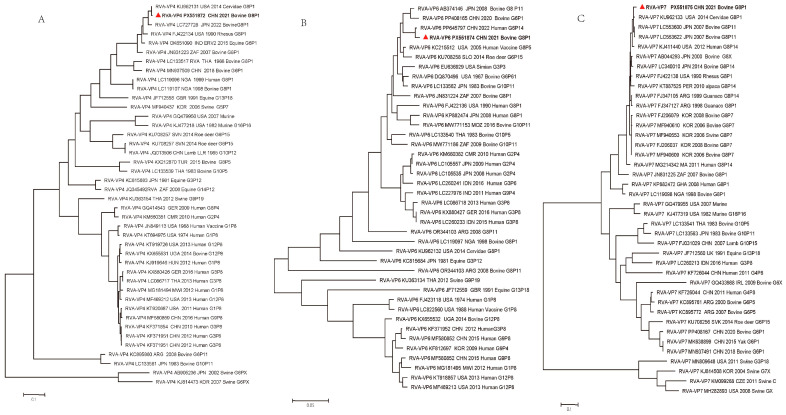
Phylogenetic trees based on the ORF sequences of VP4, VP6, and VP7 genes. (**A**) Phylogenetic tree based on the VP4 ORF sequence (2331 bp). The strain from this study is indicated with a red triangle and corresponds to accession number PX551872. (**B**) Phylogenetic tree based on the VP6 ORF sequence (1194 bp). The strain from this study is indicated with a red triangle and corresponds to accession number PX551873. (**C**) Phylogenetic tree based on the VP7 ORF sequence (981 bp). The strain from this study is indicated with a red triangle and corresponds to accession number PX551874. The BRV-YN1-2021 isolate from this study is marked with a red triangle in all panels. Trees were constructed using the maximum likelihood method with 1000 bootstrap replicates. Bootstrap values are shown at branch nodes. GenBank accession numbers, host species, countries of origin, and genotypes are indicated for each reference strain.

To establish the genetic relationships between the BRV-YN1-2021 strain and previously reported reference RVA strains, phylogenetic trees were constructed based on the open reading frame (ORF) sequences of the VP4, VP6, and VP7 gene segments. The analysis included 41 reference strains for the VP4 and VP7 genes and 39 reference strains for the VP6 gene (Figure 2A–C).

The VP4 gene of BRV-YN1-2021 exhibited the closest relationship with the cervid rotavirus strain C/Cervidae/United States/14-02218-2/2014/G8P[1] isolated in the USA (Figure 2A) and the bovine isolate RVA/Bovine-tc/JPN/AH1041/2022/G8P[1] (LC727728) from Japan. Phylogenetic analysis of the VP4 gene also demonstrated that the most closely related Chinese isolate was RVA/Cow-tc/CHN/SDC1/2018/G6P[1], which was isolated from a cow in China. The VP6 gene of BRV-YN1-2021 belonged to the I2 genotype and was closely related to several species strains, including the human strain RVA/Human-tc/CHN/MY614142/2022/G6P[14] (PP645797), the bovine strain RVA/Cow/CHN/2020/G6P[1] (PP408165), and the bovine strain RVA/Bovine/Sun9/Japan/2008/G8P[11](AB374146) (Figure 2B). The VP7 gene of BRV-YN1-2021 clustered closely with the cervid strain C/Cervidae/USA/14-02218-2/2014/G8P[1] (KU962133) and the bovine rotavirus strain RVA/Cow-tc/JPN/GB20-25/2007/G8P[11] (LC553622) (Figure 2C).

### 3.6. Pathogenicity of BRV Strain BRV-YN2021 in Suckling Mice

**Figure 3:**
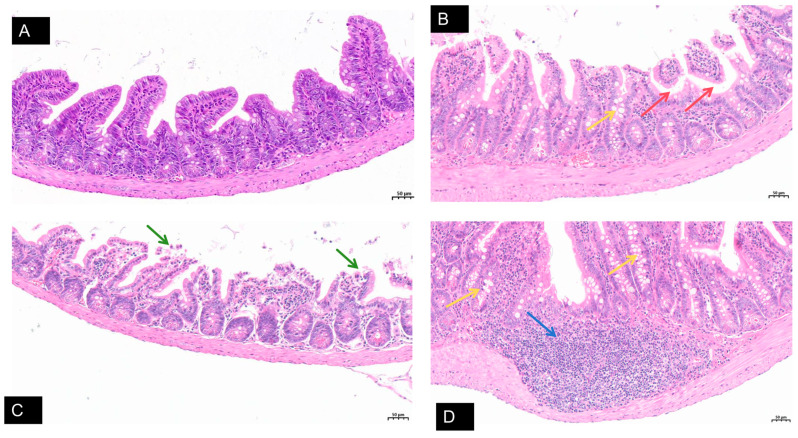
Histopathological changes in the small intestine of suckling mice experimentally infected with bovine rotavirus (H&E staining). (**A**) Control group showing normal intestinal tissue structure with intact mucosal villi and tightly arranged lamina propria, free of obvious lesions. (**B**,**C**) Infected group showing loosely arranged villous epithelial cells, partial villous detachment (red arrows), increased goblet cells (yellow arrows), degeneration, lysis, and necrosis of intestinal epithelial cells (green arrows), as well as edema of the lamina propria. (**D**) Infected group showing extensive inflammatory cell infiltration, predominantly lymphocytes and neutrophils, within the lamina propria of the mucosal layer (blue arrows). Scale bar = 50 µm (applies to all panels).

Mice infected with BRV-YN1-2021 exhibited pronounced clinical symptoms, including diarrhea and loss of appetite, whereas control mice displayed no similar clinical signs throughout the observation period. Histopathological examination of intestinal tissues revealed marked differences between the infected and control groups (Figure 3). The control group exhibited normal intestinal histomorphology with an ordered villous structure and intact epithelial lining (Figure 3A). In contrast, mice infected with BRV-YN1-2021 showed significant pathological changes in the intestinal mucosa. These alterations included loosely arranged villous epithelial cells, partial shedding of virus-infected cells (red arrows), and an increased number of goblet cells (yellow arrows). Intestinal epithelial cells exhibited degeneration, dissolution, and necrosis, accompanied by prominent submucosal edema (Figure 3B,C). Additionally, a substantial inflammatory cell infiltrate, composed predominantly of lymphocytes and neutrophils, was observed within the submucosal layer (blue arrows) (Figure 3D).

These findings demonstrate that BRV-YN1-2021 is capable of inducing significant intestinal pathology in suckling mice, characterized by epithelial damage, goblet cell hyperplasia, and inflammatory infiltration, consistent with acute viral enteritis.

## 4. Discussion

Bovine diarrhea is a significant disease that causes considerable harm to the global cattle industry [27]. Bovine RVA is the most common pathogen causing acute diarrhea in calves and is also considered a potential pathogen for acute diarrhea in other animals and humans [28].

In China, rotavirus is a major pathogen for calf diarrhea, leading to substantial economic losses due to increased mortality rates, higher treatment costs, and reduced weight gain in affected cattle [29]. Hence, epidemiological studies are of great importance to assess the impact of RVA infections on livestock health. In this study, RVA was detected at three farms during NCD outbreaks. The epidemiological investigation of RVA infections in diarrheic calves (21.5%) was similar with previous studies conducted in Australia (31%) [30], Brazil (26.2%) [31], and Sweden (22.6%) [28].

Two G genotypes (G6 and G10) circulate widely among dairy calves in China [32], with G6P[1] being the dominant genotype [18]. It is still unknown which factors determinate the differential distribution of G/P genotypes in cattle. However, the exchange of breeding bulls in different farms and regions is highly suspicious. Pathogenesis studies of BRV indicate that the G6P[1] genotype remains the dominant strain in countries neighboring with China; however, there have been no infections of rotavirus genotype G8P[1] reported in ruminants in China. The G8 genotype of rotavirus was first detected in acutely infected children in Japan in 1994 [33]. Genetic typing studies revealed that the strain underwent reassortment with an unidentified ruminant-derived strain. In China, a nationwide rotavirus survey in hospital infection revealed that G9P[8] was the dominant genotype, accounting for 79.6%, followed by G8P[8] at (13.0%) [34]. It indicates that the G8P genotype was distributed in the population. The earliest report of cattle G8P[1] was reported in Thailand in 1988. Analysis of four prevalent strains of bovine RVA indicated that two of them were G8P[1] [35]. In addition, the pathogen research conducted in Japan in 2019 revealed one isolate had a constellation of the G8P[1] genotype [36]. For other small ruminant diarrhea, research from Turkey has shown that sheep can also be infected by G8P[1] genotypes, resulting in severe economic losses [37]. The prevalence of bovine RVA in this study aligns with the rates reported in China’s literature [20]. Among the RVA-positive calves, four fatalities were directly attributed to diarrhea-related complications, emphasizing the urgent need for effective control strategies to address BRV’s impact on calf health and farm productivity.

The isolates of Yunnan’s prevalent strain belong to the G8P[1] genotype, exhibiting a distinct genetic evolutionary relationship with other bovine rotavirus isolates in China. We assembled and classified all 11 genomic segments of the BRV strain and constructed a mega-phylogenetic tree based on the VP4, VP6 and VP7 genes for evolutionary analysis. Genomic composition and phylogenetic evidence indicate that the emergence of this strain is likely driven by multiple reassortment events and interspecies transmission between humans and animals. Specifically, the strain appears to have originated from several reassortments involving bovine, deer, and human rotaviruses, supported by high sequence similarity between its genomic segments and corresponding segments from known human and deer rotaviruses. Notably, studies have confirmed that G8 rotavirus is a common circulating strain among infants and young children in China [38], consistent with the genotype identified in this study. Further genomic analysis revealed that genes 1 (VP1), 3 (VP3), 6 (VP6), 8 (NSP2), and 10 (NSP4) show extremely high sequence identity to their counterparts in human rotavirus strains. Phylogenetic results support the hypothesis, proposed earlier by Esona and Matthijnssens [39], of a common evolutionary origin for G8 human rotaviruses and bovine rotaviruses [40]. The surveillance of G/P types among bovine RVA field strains is important because it allows for the discovery of new or emerging strains and provides insights into the epidemiology of animal infections which may represent interspecies transmission events [41]. The phylogenetic relationship revealed the reassortment of a bovine RVA strain with human and deer RVA strains, specifically involving the VP4 genotype G8P[1] in China. In this survey, isolate BRV-YN1-2021 has almost certainly evolved in parallel with a USA human and deer G8P[1] strain. These findings regarding a novel strain and recombination suggest the necessity of an increased understanding of RVA ecology, epidemiology, and evolution at regional and global levels. This will require continuous molecular epidemiological surveillance from a worldwide perspective, along with comprehensive molecular characterization of BRV circulating in livestock and wildlife reservoirs. The knowledge gained from such efforts can aid the detection of newly emerging strains, helping to confirm the ongoing efficacy of human vaccine programs. However, the oral rotavirus vaccine, covering genotypes G1, G2, G3, G4, G8, and G9, is already recommended for children in China. A commercial vaccine for animals remains unavailable in China but the G6(IV)P[5] strain (UK) has been commercialized for cattle in some countries [42]. We think that the G8 genotype is also expected to be included in immunization programs for future animal vaccine development, thereby reducing suffering and economic losses.

Suckling mice are frequently used to model viral diseases. The selection of newborn mice is primarily due to their distinct physiological and immunological characteristics, which make them a more suitable model, and suckling mice are often more susceptible to infection. Many viruses, such as enterovirus and arboviruses, replicate to higher titers in the tissues of newborn mice and high viral load is crucial for pathogenicity studies [43,44]. A neonatal mouse model of rhesus rotavirus (RRV) was used to examine the mechanism of the extraintestinal spread of viruses following oral inoculation. The spread-competent viruses, RRV and reassortant R7, demonstrated a temporal progression from the intestine, to the terminal ileum, to the mesenteric lymph nodes (MLN), and to the peripheral tissues [45]. Since establishing a bovine infection model is difficult, we chose suckling mice, based on the previously described models, to perform the pathological analysis of rotavirus isolates.

RVA infections are still a frequent and important cause of diarrhea in neonatal calves and can lead to great losses on cattle farms. The identification of RVA genotypes circulating on the outbreak farm provides critical baseline data for informing targeted interventions. Genomic analyses revealed potential genetic relationships between bovine and human rotavirus strains, emphasizing the need for ongoing surveillance to monitor interspecies transmission. The ML analysis of BRV-YN1-2021 sequences revealed that bovine RVA strains, including G6P[1] and G8P[1], can infect humans either directly or through reassortment with human RVA strains, underscoring the zoonotic potential of bovine rotaviruses.

## 5. Conclusions

Understanding the genetic diversity and evolutionary dynamics of BRV is essential for developing effective control strategies to mitigate economic losses and address potential public health risks associated with interspecies transmission. In this study, we report the first isolation of a G8P[1] bovine rotavirus strain (BRV-YN1-2021) associated with a severe diarrhea outbreak in calves from Yunnan, China, with a prevalence of 21.5%. Genomic characterization revealed a unique constellation (G8-P1-I2-R2-C2-M2-A3-N2-T6-E2-H3) resulting from multiple reassortment events involving bovine, deer, and human rotaviruses. Five genomic segments showed high identity with human strains, while four segments were closely related to deer strains, providing evidence for interspecies transmission. The isolate demonstrated pathogenicity in suckling mice, inducing characteristic diarrhea and intestinal histopathology. These findings highlight the emergence of a reassortant strain with zoonotic potential and emphasize the need for continuous molecular surveillance of rotaviruses in cattle populations.

## Figures and Tables

**Table 1:** Genomic features, sequence identity analysis, and related reference strains of the BRV-YN1-2021 isolate.

Protein	Length bp (This Study)	Genotype	Nucleotide Percent Identity with Complete CDS Among 100 Sequences Selected (Blast)	Amino Acid Percent Identity with 100 Sequences Selected	Closest Strain	Host	Country	Nucleotide Sequence Accession Numbers
VP7	981	G8	96.3–97.7	71.17–99.1	RVA/Cow-tc/JPN/GB12-22/2007/G8P[11]	Cow	Japan	LC553600
VP4	2331	P[1]	75.5–99.7	57.0–99.7	C/Cervidae/United States/14-02218-2/2014	Cervidae	USA	KU962131
VP6	1194	I2	95.1–99.3	52.1–100.0	RVA/Human-tc/CHN/MY614142/2022/G6P[14]	Human	China	PP645797
VP1	3267	R2	93.8–97.2	81.9–98.6	RVA/Human-wt/JPN/Ni17-46/2017/G15P[14]	Human	Japan	MW292229
VP2	2643	C2	92.8–99.6	43.8–100.0	C/Cervidae/United States/14-02218-2/2014	Cervide	USA	KU962129
VP3	2507	M2	92.0–96.7	41.8–96.8	RVA/Human-wt/USA/3000015004/2014/G6P[14]	Human	USA	OP680461
NSP1	1476	A3	91.1–99.2	50.7–99.8	C/Cervidae/United States/14-02218-2/2014	Cervidae	USA	KU962134
NSP2	953	N2	93.1–98.7	33.2–99.8	RVA/Human-tc/VEN/RRV_NB1215_31/1987/G3P[3]	Human	Venezuela	LC438930
NSP3	901	T6	93.2–99.5	46.5–98.5	C/Cervidae/United States/14-02218-2/2014	Cervidae	USA	KU962136
NSP4	527	E2	91.5–97.5	49.7–97.9	RVA/Human-wt/US/2012841174/2012/G8P[14]	Human	USA	KJ411441
NSP5	557	H3	95.5–99.8	29.8–100	RVA/Cow-tc/China/SCMY1/2021	Cow	China	ON012973

**Table 2:** Whole-genome genotype classification of bovine rotaviruses included in this study and representative reference strains from GenBank.

Rotavirus Strain Names	Genome Constellation										
	VP7	VP4	VP6	VP1	VP2	VP3	NSP1	NSP2	NSP3	NSP4	NSP5
This study BRV-YN1-2021_RVA/Yunnan China/Bovine/G8P[1]	G8	P1	I2	R2	C2	M2	A3	N2	T6	E2	H3
RVA/Cow-tc/USA/WC3/1981/G6P[5]	G6	P5	I2	R2	C2	M2	A3	N2	T6	E2	H3
RVA/Cow-wt/ZAF/1604/2007/G8P[1]	G8	P1	I2	R2	C2	M2	A3	N2	T6	E2	H3
C/Cervidae/United States/14-02218-2/2014	G8	P1	I2	R2	C2	M2	A3	N2	T6	E1	H3
RVA/Human-wt/GHA/Ghan-059/2008/G8P[1]	G8	P1	I2	R2	C2	M2	A11	N2	T6	E2	H3
RVA/Human-wt/COD/DRC86/2003/G8P[6]	G8	P6	I2	R2	C2	M2	A2	N2	T2	E2	H2
RVA/Human-tc/USA/DS-1/1976/G2P1B4	G2	P4	I2	R2	C2	M2	A2	N2	T2	E2	H2
RVA/Cow-tc/THA/61A/1989/G10P[5]	G10	P5	I2	R2	C2	M2	A3	N2	T6	E2	H3
RVA/Cow-tc/JPN/KK3/1983/G10P[11]	G10	P11	I2	R2	C2	M2	A13	N2	T6	E2	H3
RVA/Human-wt/USA/SSCRTV00005/2013/G12P[8]	G12	P8	I1	R1	C1	M1	A1	N1	T1	E1	H1
RVA/Human-wt/BEL/B4633/2003/G12P[8]	G12	P8	I1	R1	C1	M1	A1	N1	T1	E1	H1
RVA/Human-tc/USA/Wa/1974/G1P[1]	G1	P8	I1	R1	C1	M1	A1	N1	T1	E1	H1
RVA/Human-wt/KOR/Seoul0373/2008/G9P[8]	G9	P4	I1	R1	C1	M1	A1	N1	T1	E1	H1
RVA/Human-tc/JPN/AU-1/1982/G3P3[9]	G3	P9	I3	R3	C3	M3	A3	N3	T3	E3	H3
RVA/Human-tc/THA/T152/1998/G12P[9]	G12	P9	I3	R3	C3	M3	A3	N3	T3	E3	H3
RVA/Pigeon-tc/JPN/PO-13/1983/G18P[17]	G18	P17	I4	R4	C4	M4	A4	N4	T4	E4	H4

Red (14-02218-2-like), blue (SSCRTV00005-like), yellow (AU-like), and green (some typical animal rotavirus genotypes).VP, viral structural protein; NSP, viral non-structural protein.

**Table 3:** BRV strain of BRV-YN1-2021 VP4 and VP7 nucleotide identity with the reference strains from GenBank database.

Strain/Host	Accession Number	Genotype	VP4(%)		Strain/Host	Accession Number	Genotype	VP7(%)	
			nt	aa				nt	aa
C/Cervidae/United States/14-02218-2/2014	KU962131	G8P[1]	99.7	99.7	RVA/Cow-tc/JPN/GB12-22/2007	LC553600	G8P[11]	97.7	99.1
RVA/Bovine-tc/JPN/AH1041/2022	LC727728	G8P[1]	96.9	99.1	C/Cervidae/USA/14-02218-2/2014	KU962133	G8P[1]	97.6	99.1
RVA/Horse-wt/IND/ERV2/2015	OK651090	G6P[1]	94.4	98.5	RVA/Cow-tc/JPN/GB20-25/2007	LC553622	G8P[11]	97.6	99.1
RVA/Rhesus-tc/USA/PTRV/1990	FJ422134	G8P[1]	94.1	98.8	RVA/Human-wt/US/2012841174/2012	KJ411440	G8P[14]	96.5	98.2
RVA/Cow-wt/ZAF/1604/2007	JN831223	G8P[1]	92.4	98.6	RVA/Rhesus-tc/USA/PTRV/1990	FJ422138	G8P[1]	96.2	97.9
RVA/Cow-tc/CHN/SDC1/2018	MN937509	G6P[1]	81.5	93.1	RVA/Bovine/JPN/Tokushima9503/2020	AB044293	G8	95.8	98.5
RVA/Human-tc/NGA/HMG035/1999	LC119096	G8P[1]	80.9	92.8	RVA/Bovine-tc/KOR/KJ11/2006	MF940609	G8P[7]	95.8	98.5
RVA/Cow-tc/THA/A5-10/1988	LC133517	G8P[1]	80.9	92.8	RVA/Guanaco-wt/ARG/Chubut/1999	FJ347105	G8P[14]	95.8	98.5
RVA/Cow-tc/NGA/NGRBg8/1998	LC119107	G8P[1]	80.7	92.5	RVA/Porcine-tc/KOR/174-1/2006	MF940553	G8P[7]	95.8	98.2
RVA/Horse-tc/GBR/L338/1991	JF712558	G13P[18]	77.2	84.3	RVA/Bovine/KOR/KJ111/2008	FJ206037	G8P[7]	95.8	96.9
RVA/Porcine-tc/KOR/K71/2006	MF940437	G5P[7]	74.6	81.9	RVA/Guanaco-wt/ARG/Rio_Negro/1998	FJ347127	G8P[1]	95.7	98.5
RVA/Horse-tc/JPN/BI/1981	KC815683	G3P[12]	74.2	80.2	RVA/Human-wt/JPN/12597/2014	LC340010	G8P[14]	95.7	97.9
RVA/Horse-wt/ZAF/EqRV-SA1/2006	JQ345492	G14P[12]	74.2	81.0	RVA/alpaca-wt/PER/562/2010	KT887525	G8P[14]	95.5	97.6
RVA/Lamb/CHN/LLR/1985	JQ013506	G10P12	73.8	81.9	RVA/Bovine-tc/KOR/KJ11/2006	MF940610	G8P[7]	95.4	97.6
RVA/Lamb/CHN/CC0812-1/2008	HQ834199	G10P[15]	73.7	81.6	RVA/Bovine/KOR/KJ1172/2008	FJ206079	G8P[7]	95.3	96.0
RVA/roe deer-wt/SLO/D38-14/2014	KU708257	G6P[15]	73.2	82.4	RVA/Human-tc/MAR/ma31/2011	MG214342	G8P[14]	93.5	98.5
RVA/Pig-wt/THA/CMP-015-12/2012	KU363154	G9P[19]	70.8	75.8	RVA/Cow-wt/ZAF/1604/2007	JN831225	G8P[1]	88.0	97.2
RVA/Cow-wt/UGA/BUW-14-A035/2014	KX655531	G12P[8]	70.1	72.9	RVA/Human-tc/NGA/HMG035/1999	LC119098	G8P[1]	84.9	96.3
RVA/Human-GER1H-09/2009	GQ414543	G8P[4]	70.1	71.9	RVA/Human-wt/GHA/Ghan-059/2008	KP882472	G8P[1]	84.3	94.5
RVA/Human-wt/DEU/GER34-16/2016	KX880426	G3P[8]	70.1	72.4	RVA/Cow-tc/JPN/KK3/1983	LC133563	G10P[11]	76.4	82.0
RVA/Human-wt/THA/SKT-281/2013	LC086717	G3P[8]	70.1	72.4	RVA/Human-wt/IDN/SOEP075/2016	LC260213	G3P[8]	76.0	84.7
RVA/Human-wt/HUN/ERN5125/2012	KJ919646	G1P[8]	70.0	72.7	RVA/Cow-tc/THA/61A/1989	LC133541	G10P[5]	76.0	82.9
RVA/Human-wt/USA/SSCRTV_00005/2013	MF469212	G12P[8]	69.8	72.7	RVA/Human-wt/AUS/CK20038/2008	KC443372	G6P[4]	75.	82.9
RVA/Human-wt/USA/VU12-13-132/2013	KT919726	G12P[8]	69.8	72.7	RVA/Cow-wt/ARG/B3035_B_BA/2007	KC895772	G6P[5]	75.1	82.6
RVA/Human-wt/CMR/BA366/2010	KM660351	G2P[4]	69.8	71.3	RVA/roe deer-wt/SLO/D38-14/2014	KU708256	G6P[15]	74.9	85.0
RVA/Human-wt/MWI/BID110/2012	MG181494	G1P[8]	69.6	72.4	RVA/Cow/CHN/DQ2020/2020	PP408167	G6P[1]	74.8	83.8
RVA/Human-wt/CHN/E2432/2010	KF371854	G3P[8]	69.6	72.2	RVA/Cow-wt/ARG/B1186_B_ER/2000	KC895761	G6P[5]	74.6	82.3
RVA/Cow-tc/THA/61A/1989	LC133539	G10P[5]	69.5	74.3	RVA/Murine/USA/EB-kk18/1982	KJ477319	G16P[16]	74.5	82.6
RVA/Human-wt/CHN/E2484/2011	KF726045	G4P[8]	69.5	72.2	RVA/Murine/USA/ETD_822/2007	GQ479955	/	74.2	83.8
RVA/Human-wt/CHN/L1450/2012	KF371951	G3P[8]	69.5	72.0	RVA/CHN/Lamb-NT/2007	FJ031029	G10P[15]	74.1	82.3
RVA/Cow-wt/TUR/Amasya-2/2015	KX212870	G8P[5]	69.3	74.6	RVA/Horse-tc/GBR/L338/1991	JF712560	G13P[18]	74.0	77.7
RVA/Human-wt/USA/CNMC125/2011	KT920687	G1P[8]	69.3	72.3	RVA/Yak-tc/CHN/QH-1/2015	MK638899	G6P[1]	73.7	82.4
RVA/Human-wt/CHN/Hu/JS/2016	MF580859	G9P[8]	69.2	72.2	RVA/Cow-wt/CHN/SDC5/2018	MN937491	G6P[1]	73.7	82.4
RVA/Human-tc/USA/Wa-40-AG/1974	KT694975	G1P[8]	68.7	71.6	RVA/Human-wt/CHN/E2484/2011	KF726044	G4P[8]	71.4	74.3
RVA/Vaccine/USA/Rotarix-A41CB052A/1988	JN849113	G1P[8]	68.7	71.4	RVA/Bovine/IRL/UCD-RVL-Bov5/2009	GQ433988	G6	65.8	81.9
RVA/Murine/USA/ETD_822/2007	GQ479950	/	68.1	76.3	RVC/Pig-wt/KOR/1027/2012	KJ814508	G7PX	49.2	33.1
RVA/Murine/USA/EB-aa40/1982	KJ477218	G16P[16]	67.8	75.0	RVC/Pig-wt/USA/RV0104/2011	MN809648	G3P18	48.7	30.0
RVA/Cow-wt/ARG/B3700_D_BA/2008	KC895860	G6P[11]	61.5	59.9	RVC/Pig-wt/CZE/P303/2011	KM099268	/	48.4	33.1
VA/Cow-tc/JPN/KK3/1983	LC133561	G10P[11]	61.3	59.7	RVC/Pig-wt/USA/OK.5.68/2008	MH282893	GX	48.2	31.6
RVA/porcine-CJ31-6/JPN/2002	AB905236	G8P[1]	50.1	32.4					
RVC/Pig-wt/KOR/07-109-12/2007	KJ814473	G6PX	49.6	32.0					

## Data Availability

The complete genome sequences of the rotavirus strains generated in this study have been deposited in the NCBI GenBank database under accession numbers PX551869–PX551879.

## Abbreviations

The following abbreviations are used in this manuscript:

BRV	Bovine rotavirus
RVAs	Group A rotaviruses
NCD	Neonatal calf diarrhea
RT-PCR	Reverse transcription polymerase chain reaction
CPE	Cytopathic effect
ORF	Open reading frame
UTR	Untranslated region
